# Biochemical and genetic dissection of the RNA-binding surface of the FinO domain of *Escherichia coli* ProQ

**DOI:** 10.1261/rna.079697.123

**Published:** 2023-11

**Authors:** Ewa M. Stein, Suxuan Wang, Katherine G. Dailey, Chandra M. Gravel, Shiying Wang, Mikołaj Olejniczak, Katherine E. Berry

**Affiliations:** 1 Institute of Molecular Biology and Biotechnology, Faculty of Biology, Adam Mickiewicz University, 61-614 Poznań, Poland; 2 Program in Biochemistry, Mount Holyoke College, South Hadley, Massachusetts 01075, USA; 3 Department of Chemistry, Mount Holyoke College, South Hadley, Massachusetts 01075, USA

**Keywords:** *Escherichia coli* ProQ, FinO-domain proteins, RNA–protein interactions, bacterial small RNAs, bacterial three-hybrid assay

## Abstract

RNA-binding proteins play important roles in bacterial gene regulation through interactions with both coding and noncoding RNAs. ProQ is a FinO-domain protein that binds a large set of RNAs in *Escherichia coli*, though the details of how ProQ binds these RNAs remain unclear. In this study, we used a combination of in vivo and in vitro binding assays to confirm key structural features of *E. coli* ProQ’s FinO domain and explore its mechanism of RNA interactions. Using a bacterial three-hybrid assay, we performed forward genetic screens to confirm the importance of the concave face of ProQ in RNA binding. Using gel shift assays, we directly probed the contributions of ten amino acids on ProQ binding to seven RNA targets. Certain residues (R58, Y70, and R80) were found to be essential for binding of all seven RNAs, while substitutions of other residues (K54 and R62) caused more moderate binding defects. Interestingly, substitutions of two amino acids (K35, R69), which are evolutionarily variable but adjacent to conserved residues, showed varied effects on the binding of different RNAs; these may arise from the differing sequence context around each RNA’s terminator hairpin. Together, this work confirms many of the essential RNA-binding residues in ProQ initially identified in vivo and supports a model in which residues on the conserved concave face of the FinO domain such as R58, Y70, and R80 form the main RNA-binding site of *E. coli* ProQ, while additional contacts contribute to the binding of certain RNAs.

## INTRODUCTION

Small RNAs (sRNAs) participate in the regulation of gene expression in bacteria, contributing to the maintenance of cellular homeostasis and adaptation to environmental changes (Wagner and Romby 2015; Gorski et al. 2017; Adams and Storz 2020). Hfq and ProQ are both global RNA-binding proteins involved in sRNA-dependent regulation in *Escherichia coli* and *Salmonella enterica*, which bind distinct, but partly overlapping RNA pools (Holmqvist et al. 2016, 2018; Melamed et al. 2016, 2020; Smirnov et al. 2016). ProQ binds hundreds of RNAs in *E. coli* and *S. enterica* (Holmqvist et al. 2018; Melamed et al. 2020), and contributes to such processes as DNA maintenance (Smirnov et al. 2016, 2017), adaptation to osmotic stress (Kerr et al. 2014; Melamed et al. 2020), motility (Rizvanovic et al. 2021), carbon source utilization (El Mouali et al. 2021), adaptation to nutrient availability (Avrani et al. 2017; Knöppel et al. 2018; Gross et al. 2020; Katz et al. 2021), and virulence (Westermann et al. 2019). While Hfq has many well-established mechanisms of action (e.g., influencing sRNA–mRNA base pairing, sRNA lifetimes, mRNA translation, and RNA folding [Moll et al. 2003; Rajkowitsch and Schroeder 2007; Soper and Woodson 2008; Fender et al. 2010; Panja et al. 2013; Updegrove et al. 2016; Chen and Gottesman 2017; Andrade et al. 2018; Kavita et al. 2018]), much less is known about the detailed mechanisms used by ProQ to bind RNA and regulate gene expression.

ProQ belongs to the FinO family of proteins. Each member contains a conserved RNA-binding FinO domain, named after the founding member of this family, the F-like plasmid (F′) FinO protein (Supplemental Figs. S1, S2; Ghetu et al. 2000; Glover et al. 2015; Olejniczak and Storz 2017; Holmqvist et al. 2020). Proteins from this family are present in numerous proteobacteria (Attaiech et al. 2017). In *E. coli* and *S. enterica* ProQ, the FinO amino-terminal domain (NTD) is connected via a long, positively charged linker to the carboxy-terminal Tudor domain (CTD) (Smith et al. 2004, 2007; Gonzalez et al. 2017). The FinO domain has been shown to be the primary RNA-binding site of ProQ in vitro and in vivo (Chaulk et al. 2011; Pandey et al. 2020; Stein et al. 2020).

Multiple approaches have been used to identify the residues of FinO-domain proteins that participate in RNA binding. For instance, the surfaces of the *E. coli* ProQ FinO domain that are protected by the binding of two RNAs, *cspE* and SraB, have been mapped using hydrogen–deuterium exchange experiments (Gonzalez et al. 2017). Crosslinking experiments in the homologous F′ FinO protein showed contacts between a FinP RNA fragment and several amino acid residues located mainly on the concave face of the FinO domain (Ghetu et al. 2002). The role of the concave face in RNA binding has also been supported by NMR studies of *Legionella pneumophila* Lpp1663 protein, which showed strong chemical shifts on the concave side of the FinO domain upon the binding of a U_6_ oligoribonucleotide or a hairpin derived from RaiZ sRNA (Immer et al. 2020). In addition, a recent cocrystal structure was solved showing how the terminator hairpin of *LP* RocR binds to the FinO domain of RocC (Kim et al. 2022). This structure joins an additional four experimentally determined structures of FinO-domain orthologs from various bacterial species (Supplemental Fig. S2; Ghetu et al. 2000; Chaulk et al. 2010; Gonzalez et al. 2017; Immer et al. 2020) and, more recently, AlphaFold predictions of these structures (Jumper et al. 2021). Several amino acids in ProQ’s FinO domain have been shown to play a role in RNA binding and function in vivo using phenotypic screening studies (El Mouali et al. 2021; Rizvanovic et al. 2021).

The cocrystal structure of the FinO domain of *L. pneumophila* RocC with the terminator hairpin of RocR revealed several features of RNA interaction with a FinO domain (Kim et al. 2022). In the structure, the amino acid residues that contact the 3′-terminal single-stranded RNA tail include the conserved arginine 97 and tyrosine 87. On the other hand, the double-stranded stem of the hairpin is recognized by amino acid residues located on the amino-terminal part of α-helix 5, termed the “N-cap motif,” which includes lysine and serine residues that interact with RNA phosphate groups. The fact that many of the features identified by this cocrystal structure involve generally conserved residues suggests that these may be recurring themes in RNA recognition by other FinO-domain proteins.

We have previously utilized a bacterial three-hybrid (B3H) assay (Berry and Hochschild 2018; Stockert et al. 2022) to detect ProQ–RNA interactions (Pandey et al. 2020; Stein et al. 2020) and screen the effects of mutations in ProQ on RNA binding in vivo. This study was the first to examine the effects of amino acid substitutions on the RNA-binding activity of *E. coli* ProQ. Both site-directed mutagenesis and unbiased screens converged on similar takeaways from this study, identifying a set of residues that contribute to SibB and *cspE* RNA binding by the FinO domain of ProQ (Pandey et al. 2020). The NMR-derived structural model available for this domain of *E. coli* ProQ (Gonzalez et al. 2017) suggested that most of the RNA-binding residues fell on the concave surface, which had been implicated in RNA binding in studies performed on other FinO-domain homologs (Ghetu et al. 2000). Intriguingly, a single residue required for RNA interaction—the highly conserved arginine 80—was modeled by the NMR structure (Gonzalez et al. 2017) to fall on the convex face of the NTD, opposite from the other RNA-binding residues on *E. coli* ProQ and from the position of homologous residues in the solved structures of other FinO domain proteins (FinO, NMB1681, Lpp1663) (Ghetu et al. 2000; Chaulk et al. 2010; Gonzalez et al. 2017; Immer et al. 2020), as well as its position on the AlphaFold structural prediction for *E. coli* ProQ (Jumper et al. 2021). The latter, however, is informed by homology so could be biased by the homologous protein structures if *E. coli* ProQ differed in this respect from other FinO domains. The location of this conserved and critical RNA-binding residue on the convex face raised the question of whether it constitutes an additional contact point in *E. coli* ProQ in addition to the concave surface.

At the end of our previous work, three primary questions remained unanswered: (i) how do we explain that the amino acid residues found to be required for RNA binding on the FinO domain of *E. coli* ProQ were quite distant from one another; (ii) will the effects of mutations observed for the few RNAs examined so far hold true for a more diverse set of RNAs bound by ProQ; and (iii) whether all of the critical residues for in vivo interactions are directly involved in RNA binding, rather than mediating indirect cellular effects?

In this work, we have used both in vivo and in vitro binding experiments to resolve these unanswered questions about ProQ–RNA interactions. We have probed the in vivo structure of the FinO domain of *E. coli* ProQ, using our B3H assay and a compensatory mutagenesis screen to shed light on the location of the conserved R80 residue. The results of this unbiased screen support a structural model in which the side chain of R80 points through to the concave face of the FinO domain, close to other RNA-binding residues and to the position of this side chain in structural homologs. Building on this structural insight, we have conducted the first in vitro site-directed mutagenesis experiments with *E. coli* ProQ to probe the contributions of both conserved and more variable amino acids on RNA binding. Gel shift and quantitative analysis of 12 ProQ mutants revealed that certain amino acids have differential contributions to binding across seven natural RNA ligands, suggesting that ProQ may utilize distinct contacts with different RNA ligands. Further, we explored the properties of RNA structure that contributed to differential interactions with ProQ’s FinO domain and demonstrated the importance of single-stranded regions both upstream and downstream from terminator hairpins. Overall, this work advances a model in which the concave face of the FinO domain serves as the main RNA-binding site of *E. coli* ProQ, and residues on the periphery of this surface tune interactions with different RNA ligands.

## RESULTS

### The concave-face pocket of the FinO domain is conserved in *E. coli* ProQ

**FIGURE 1.**
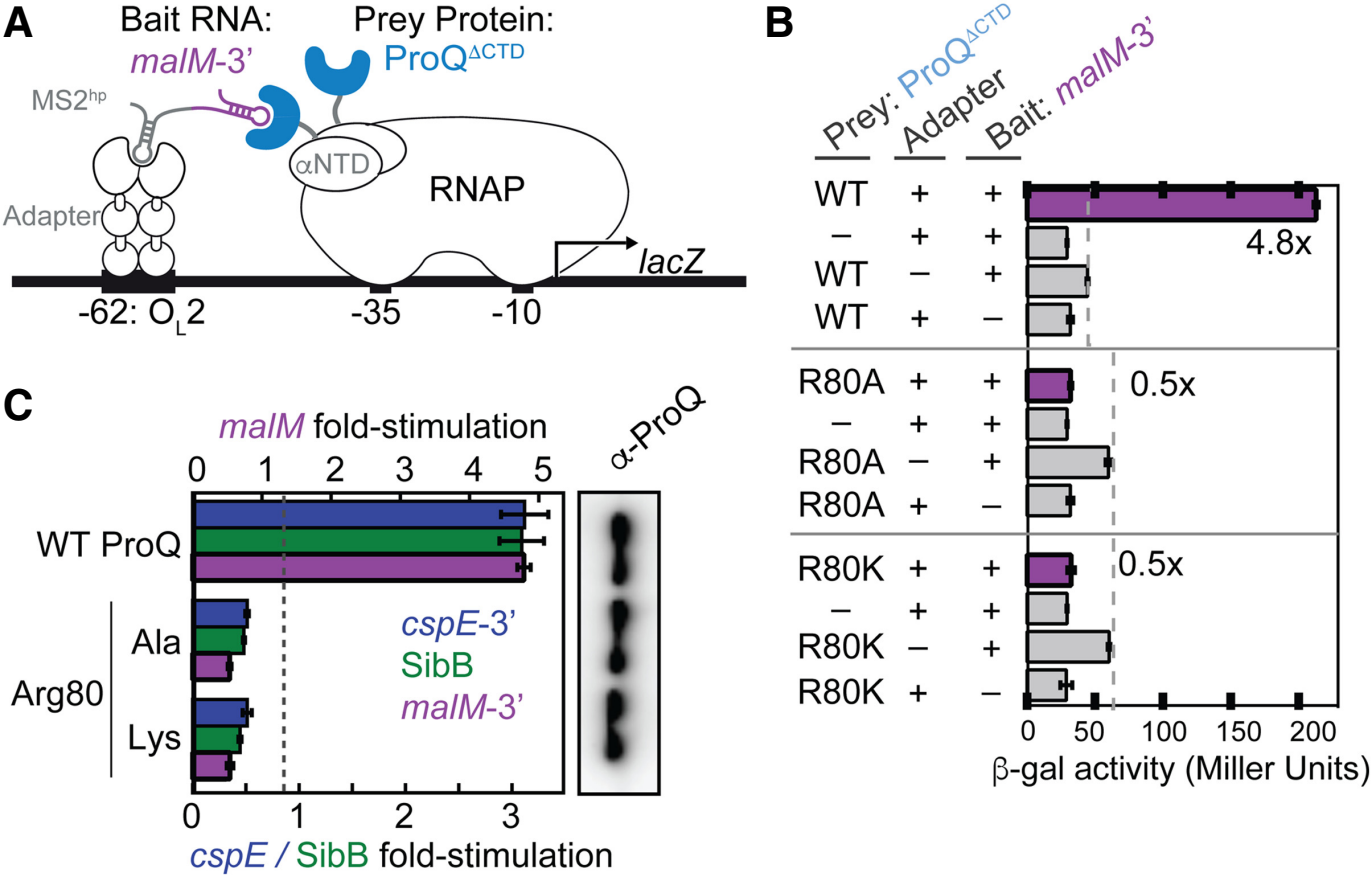
A positive charge at conserved position 80 is not sufficient for RNA interaction in vivo or in vitro. (*A*) Design of B3H system to detect the interaction between ProQ and an RNA (*malM* 3′UTR). Interaction between the protein moiety and RNA moiety fused, respectively, to the NTD of the alpha subunit of RNA polymerase (RNAP) (α) and to one copy of the MS2 RNA hairpin (MS2^hp^) activates transcription from test promoter, which directs transcription of a *lacZ* reporter gene (Berry and Hochschild 2018). Compatible plasmids direct the synthesis of the α-fusion protein (under the control of an IPTG-inducible promoter), the CI-MS2^CP^ adapter protein (under the control of a constitutive promoter; pCW17) and the hybrid RNA (under the control of an arabinose-inducible promoter). (*B*) Results of β-galactosidase (β-gal) assays performed in Δ*hfq* p*lac*-O_L_2–62 reporter strain cells containing three compatible plasmids: one (α-ProQ) that encoded α (−) or the α-ProQ^ΔCTD^ (pKB955; residues 2–176) fusion protein (WT or an R80A or R80K mutant), another (CI-MS2^CP^) that encoded λCI (−) or the λCI-MS2^CP^ fusion protein (+), and a third (Bait) that encoded a hybrid RNA with the 3′-UTR of *malM* (pKB1210) following one copy of an MS2^hp^ moiety (+) or an RNA that contained only the MS2^hp^ moiety (−). Cells were grown in the presence of 0.2% arabinose and 50 µM IPTG (see Materials and Methods). Bar graphs show the averages of three independent measurements and standard deviations. (*C*) (*Left*) Results of B3H assays detecting interactions between α-ProQ^ΔCTD^ and three RNA baits. β-gal assays were performed as in (*B*) but with three-hybrid RNA constructs (MS2^hp^-*malM*-3′, MS2^hp^-*cspE*-3′, MS2^hp^-SibB). The bar graph shows the fold-stimulation over basal levels as averages and standard deviations of values collected from three independent experiments conducted in triplicate across multiple days. (*Right*) Western blot to compare steady-state expression levels of mutant α-ProQ^ΔCTD^ fusion proteins. Lysates were taken from the corresponding β-gal experiment containing MS2^hp^-*malM-*3′ and all other hybrid components at 50 µM IPTG. Following electrophoresis and transfer, membranes were probed with anti-ProQ antibody. See Supplemental Figure S3 for loading controls and quantification of results.

We previously used an in vivo B3H assay to examine the effects of mutations on ProQ–RNA interactions (Fig. 1A; see Materials and Methods for detailed description). One mutation that caused very strong defects in RNA interaction was R80A. To further explore the role arginine 80 plays in RNA binding, and whether its role goes beyond electrostatics, we created an R80K substitution, replacing arginine with the other basic residue, lysine. We tested RNA interactions of both R80A and R80K ProQ variants using our B3H assay with three RNA ligands: *cspE*-3′, SibB, and *malM*-3′. Surprisingly, even the very conservative substitution of arginine to lysine at position 80 eliminated RNA binding to a similar extent as an alanine for all RNAs tested (Fig. 1B,C), even as western blotting showed that all three variants of α-ProQ were expressed to similar extents (Fig. 1C; quantification and loading controls in Supplemental Fig. S3).

The inability of lysine to substitute for arginine at residue 80 is analogous to the strongly deleterious effect we previously observed of a conservative Y70F substitution on RNA binding in vivo (Pandey et al. 2020). We wondered whether the positive charge and aromatic ring were not the critical features of this particular arginine and tyrosine, respectively. To explicitly test whether any other amino acid could support RNA binding at either position 70 or 80 in the FinO domain of *E. coli* ProQ, we constructed saturation mutagenesis libraries at each of these positions and screened for colonies showing any amount of blue over negative controls with *malM*-3′ RNA as bait in the B3H assay. Sequencing results of isolated plasmids showed that all codons encoding arginine at position 80 and tyrosine at position 70 were recovered (Supplemental Table S1), but no other substitutions at either position were found to support detectable binding in vivo. This confirms that both Y70 and R80 are uniquely required for RNA interaction. Indeed, these residues are both highly conserved across FinO-domain sequences and are located near one another in a concave pocket in most solved structures of FinO domains (Supplemental Figs. S1, S2).

**FIGURE 2.**
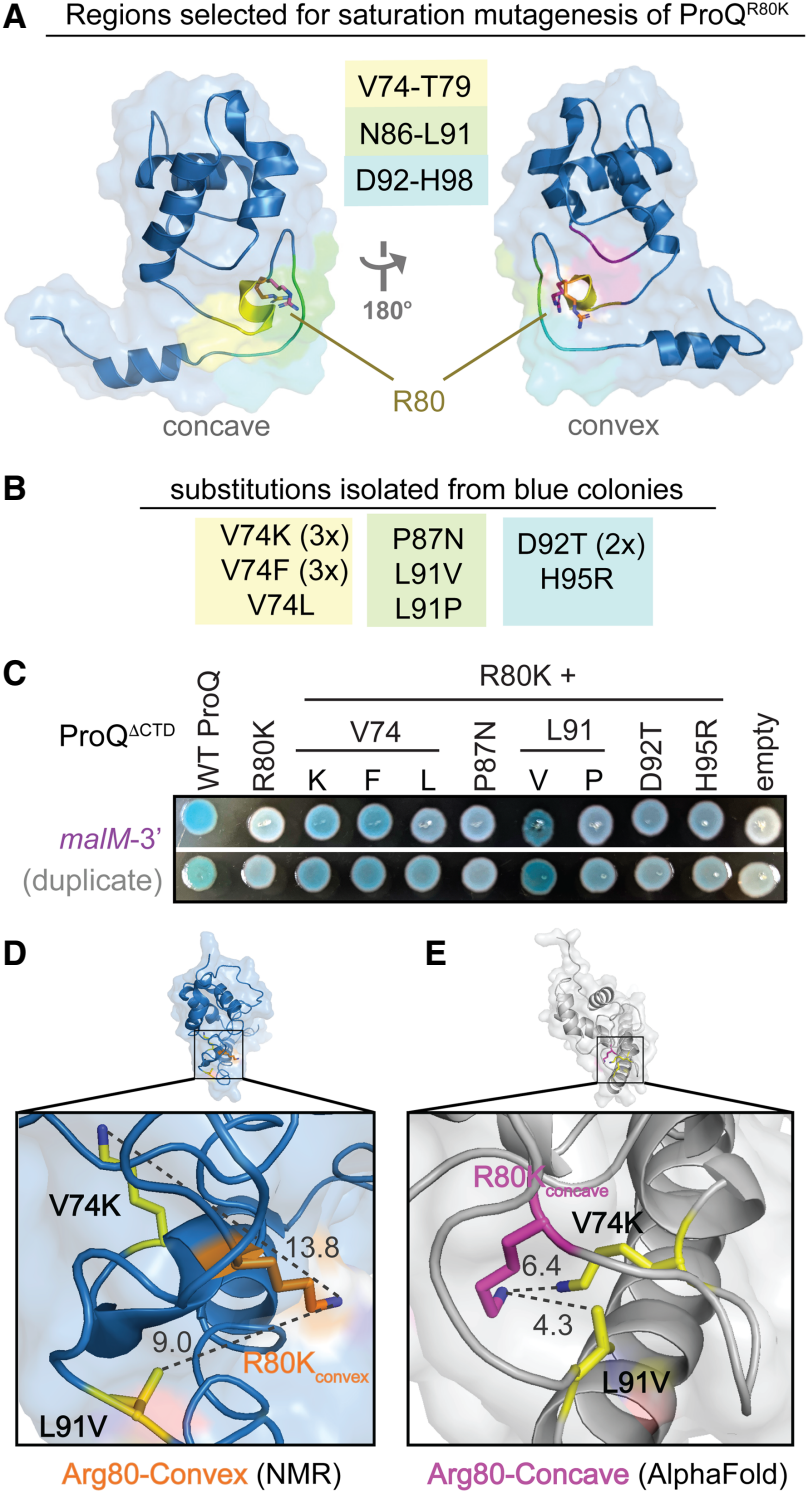
Saturation mutagenesis screen to identify compensatory mutants that rescue RNA-binding activity of ProQ^R80K^. (*A*) Residues selected for saturation mutagenesis are highlighted in yellow, green, and cyan on the NMR structure of ProQ^NTD^ (Gonzalez et al. 2017). Arginine 80 is shown in orange sticks in the convex-face position found in the NMR structure and in pink sticks in a hypothetical concave-face position analogous to its position in other orthologs. (*B*) Compensatory mutations found in pPrey-ProQ^R80K^ plasmids that produced blue colonies on X-gal-indicator plates with pBait-*malM-*3′ are listed. Codons identified in multiple colonies are indicated by 2× and 3×. (*C*) Results confirming the effects of compensatory mutants in a plate-based B3H assay, detecting interactions between variants of ProQ^ΔCTD^ with *malM-*3′ RNA. β-gal assays were performed with Δ*hfq* reporter strain cells containing three compatible plasmids: one that encoded the CI-MS2^CP^ fusion protein, another that encoded α or an α-ProQ^ΔCTD^ fusion protein (wild-type, WT, or the indicated mutant), and a third that encoded a hybrid RNA (MS2^hp^-*malM*-3′) (see Materials and Methods). Quantification of color intensity shown in Supplemental Figure S4B. (*D*,*E*) The two strongest compensatory substitutions (V74K and L91V) are shown on (*D*) the NMR structure and (*E*) the AlphaFold Model (Varadi et al. 2022) of ProQ^NTD^. Side chains were mutated in PyMol to the lowest-energy rotamer to show predicted structures of the compensatory substitutions. Distances between the terminal atom of each side chain (R80K, V74K, L71V) are shown in Angstroms and visualized with dashed lines.

Given that both Y70 and R80 were important for *E. coli* ProQ’s interaction with RNA, it was important to clarify if these residues are indeed located on opposite faces of the protein as previously suggested (Gonzalez et al. 2017; Pandey et al. 2020), especially because these residues are found on the same face of the FinO domain in the structures of other homologs (Supplemental Fig. S2; Ghetu et al. 2000; Chaulk et al. 2010; Immer et al. 2020; Kim et al. 2022). We reasoned that the chemical change in ProQ^R80K^ was minor enough that it should be possible to rescue with compensatory mutations of nearby residues—and that the location of these compensatory substitutions would provide evidence regarding the likely structural position for R80 in the structure of *E. coli* ProQ found in vivo. To create compensatory mutations that could rescue RNA binding of ProQ^R80K^, we selected three regions of the primary sequence that were close to the proposed positions of R80 in the NMR structure of *E. coli* ProQ (convex face) or in other FinO domain proteins (concave face; Fig. 2A; Supplemental Fig. S2). We then used a saturation mutagenesis strategy (Supplemental Fig. S4A) to create a library of all possible single point-mutations at each of these amino acid positions on the pPrey-ProQ^R80K^ plasmid.

To screen for compensatory mutations that could partially rescue RNA binding of ProQ^R80K^, saturation mutagenesis libraries were transformed into B3H reporter cells containing pAdapter and pBait-*malM* plasmids. Transformants were plated on X-gal-indicator plates, and plasmids were purified and sequenced from colonies that were more blue than a pPrey-empty control. Mutations that partially rescued ProQ^R80K^-*malM* binding were identified from each of the three mutagenesis libraries constructed (Fig. 2B); multiple mutations were identified at the same amino acid positions (V74 and L91) and several of the mutant plasmids were isolated and sequenced multiple times. When isolated plasmids were retransformed into fresh B3H reporter cells, many of the initial hits showed above-background interaction with *malM*-3′ RNA in qualitative plate-based assays (Fig. 2C); substitutions at V74 and L91 produced statistically significant effects when the colors from plate-based phenotypes were quantitatively analyzed (Supplemental Fig. S4B). The strongest of these substitutions also resulted in statistically significant but modest rescue of ProQ^R80K^ binding to *malM-3*′ in liquid β-gal assays (L91V, V74K; Supplemental Fig. S4C) and to *cspE-3*′ and SibB RNAs in plate-based assays (Supplemental Fig. S4D,E). The fact that compensatory effects of substitutions on RNA binding were more evident in plate-based assays with *malM*-3′ than in liquid β-gal assays or interactions with other RNA ligands is not surprising since they were identified in a plate-based screen with *malM*-3′ RNA and the conditions of the plate-based assay can be tuned to highlight small differences in β-gal levels (Stockert et al. 2022).

We next mapped the positions of the strongest confirmed compensatory substitutions to two available structures for the ProQ^NTD^ (Gonzalez et al. 2017; Jumper et al. 2021; Varadi et al. 2022). While the two models share an overall fold of the FinO domain, they differ in the region containing R80. V74 and L91, the sites of the strongest compensatory mutations, point away from the convex-facing R80 in the NMR structure (Fig. 2D; Gonzalez et al. 2017), but toward R80 and the concave face in the AlphaFold structural model (Fig. 2E; Jumper et al. 2021; Varadi et al. 2022); D92 and H95, which showed qualitative rescue of *malM-3*′ binding (Fig. 2C), further form a pocket with V74 and L91 around R80 in the AlphaFold model (Supplemental Fig. S5). Finally, when V74K and L91V substitutions are modeled into the respective structures as lowest-energy rotamers, the terminal atoms are 9–14Å away from that of a convex-facing R80K (Fig. 2D), but only 4–7Å away in the AlphaFold model (Fig. 2E). Indeed, the AlphaFold model rationalizes why the conservative R80K would be such a deleterious substitution: lysine’s shorter aliphatic side would pull the terminal polar/charged functional group into the hydrophobic core of the protein, disrupting the overall fold (Supplemental Fig. S5). The model also explains why a V74K substitution would rescue a lysine at position 80, as it provides a second amine in hydrogen-bonding distance of R80K in an otherwise hydrophobic environment (Fig. 2E).

Together, these results lend experimental validation to the AlphaFold model of *E. coli* ProQ’s FinO domain, in which all critical RNA-binding residues, including R80, are located on the concave face of the FinO domain (Fig. 2E; Supplemental Fig. S2). In this model, the concave face of the FinO domain looks quite like other structural homologs, with the highly conserved Y70 and R80 residues positioned near one another in a concave pocket.

### Central concave-pocket residues are essential for RNA binding in vitro

With this refined structural model for *E. coli* ProQ^NTD^ in mind, we wished to further explore how specific FinO-domain residues contribute to RNA binding. In particular, we wanted to test whether conclusions from B3H studies with *cspE*-3′ and SibB RNAs about the importance of specific residues on RNA binding (Pandey et al. 2020) would also hold true with purified components and for other RNA ligands of ProQ. For this purpose, we compared RNA binding to ProQ^NTD^ mutants using electrophoretic mobility shift assays, as previously conducted (Stein et al. 2020). We selected seven RNAs for this study, each of which had been found to bind to ProQ in vivo (Supplemental Fig. S6; Holmqvist et al. 2018; Melamed et al. 2020). These RNAs included *malM*-3′, the top RNA ligand identified by RIL-seq of ProQ (Melamed et al. 2020), two versions of *cspE* 3′-UTR (a 52-nt fragment [*cspE*-3′] and an 81-nt fragment [*cspE*81-3′]), *gapA*-3′, SibA and SibB, all of which are specific ligands of ProQ in vivo, and RybB, which is bound by both ProQ and Hfq in vivo. With the exceptions of SibB and RybB, the binding of all these RNAs had already been studied with purified ProQ^NTD^ (Stein et al. 2020).

**FIGURE 3.**
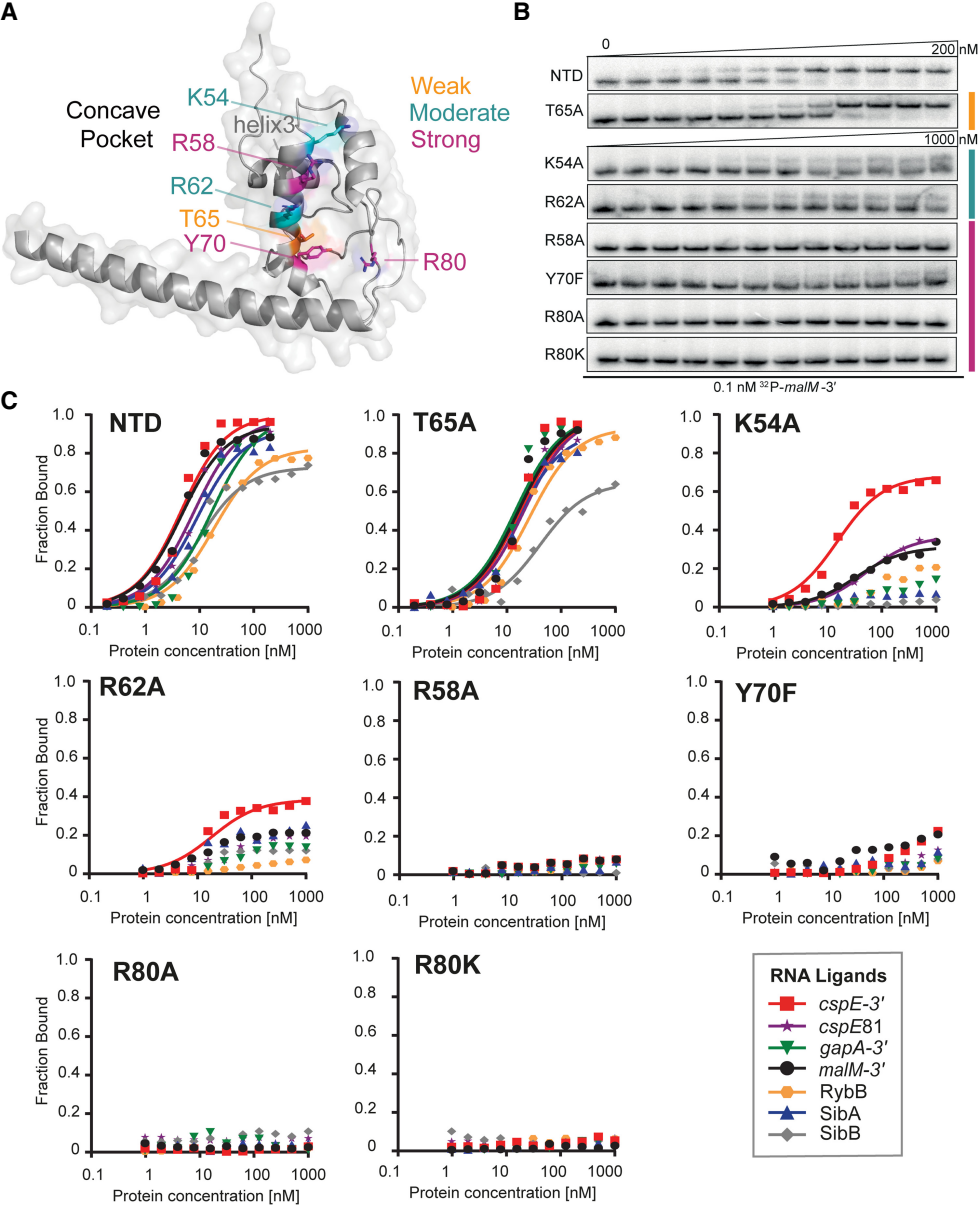
Mutations in the central pocket have strong detrimental effects on the binding of seven RNAs. (*A*) The location of substitutions on the AlphaFold model of ProQ^NTD^ (Varadi et al. 2022). (*B*) The raw gel data for 1 nM ^32^P-labeled *malM*-3′ binding to the eight proteins. (*C*) The plots of fraction bound versus protein concentration for the binding of *cspE*-3′, *cspE*81-3′, *gapA*-3′, *malM*-3′, RybB, SibA, and SibB RNAs to WT ProQ^NTD^, and T65A, K54A, R62A, R58A, Y70F, R80A, and R80K mutants measured using the gel shift assay. The data sets in which the maximum fraction bound was above 40% were analyzed by fitting to the quadratic equation. Raw gel data for all RNAs are presented in Supplemental Figures S7–S13. The obtained *K*_d_ values are shown in Table 1.

For binding studies, we used a 130-aa long version of ProQ’s NTD, the RNA-binding properties of which had been previously studied (Chaulk et al. 2011; Pandey et al. 2020; Stein et al. 2020). We first compared the binding affinities of the seven RNA ligands with WT ProQ^NTD^. The observed *K*_d_ values for ProQ^NTD^ binding to *cspE*-3′, *cspE*81-3′, *malM*-3′, SibA, and SibB fell within in a similar range (4–10 nM); only *gapA*-3′ and RybB bound weaker with *K*_d_ values of 14 nM and 26 nM, respectively (Table 1; Figs. 3; Supplemental Figs. S7–S13). Overall, the seven RNAs bound WT ProQ^NTD^ with a relatively narrow range of affinities.

**TABLE 1.**
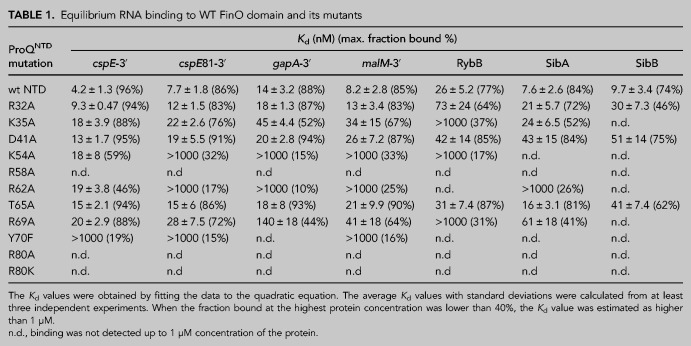
Equilibrium RNA binding to WT FinO domain and its mutants

Because the in vivo B3H data showed the importance of universally conserved residues tyrosine 70 and arginine 80 for the binding of *cspE-*3′, *malM* 3′, and SibB (Figs. 1, 2; Pandey et al. 2020), we next compared the effects of mutations at these positions on the binding of the seven RNAs. The Y70F mutant had a dramatic effect on RNA binding across all RNAs, with the fraction of RNA bound reaching no higher than 20% at the highest concentration of ProQ^NTD^ (1 µM) for *cspE*-3′, *cspE*81-3′, and *malM*-3′ RNAs, and no binding detected at all for *gapA*-3′, RybB, SibA, and SibB RNAs (Table 1; Fig. 3; Supplemental Figs. S7–S13). Because this and subsequent mutations affected the maximum fraction of RNA bound to ProQ, Table 1 reports the maximum fraction bound for each mutant as well as *K*_d_ values for experiments where the fraction bound reached at least 40%. Substitutions at R80 were even more deleterious than those at Y70: no binding was detected for any of the seven RNAs to either R80A or R80K mutants (Table 1; Fig. 3B,C; Supplemental Figs. S7–S13), consistent with the strong effect of both mutants in vivo (Fig. 1C). The fact that substitutions of Y70 and R80 were so deleterious for the binding of all seven RNAs supports the conclusion that these amino acid residues are universally important for the binding of natural RNA ligands of ProQ.

To test if the compensatory effects of double mutations observed in the B3H assay can also be observed with an in vitro binding assay, we constructed and purified the R80K-L91V double mutant, which showed restoration of binding to *malM*-3′ in both plate-based and liquid B3H assays (Fig. 2C; Supplemental Fig. S4B,C). However, we did not observe improvement in binding compared to the single R80K mutant (Supplemental Fig. S14). This suggests that despite an observable effect in the in vivo B3H assay, the compensatory role of the L91V substitution is too subtle to be detected using a gel shift assay. The lack of an effect could reflect that the complex is not sufficiently stable during the time required for electrophoretic separation. Alternatively, it is possible that the double mutant ProQ might be stabilized inside the cell by interactions with cellular RNAs or an additional factor that could support its function in the three-hybrid assay.

Next, we analyzed the role of threonine 65, which is close to Y70 on the concave face of the FinO domain (Fig. 3A). While T65 was not identified in our in vivo mutagenesis screens (Pandey et al. 2020), we hypothesized that it could be important for RNA binding based on its proximity to the side chain of Y70 as well as its conservation as a threonine or a structurally similar serine or cysteine (Supplemental Fig. S1). Despite these factors, the T65A mutation had rather small effects on RNA binding by ProQ^NTD^, ranging from no effect on the binding of *gapA*-3′ and RybB, to twofold weaker binding than WT ProQ^NTD^ for *cspE*81-3′, *malM*-3′, and SibA, and fourfold weaker binding for *cspE*-3′ and SibB (Table 1; Fig. 3B,C; Supplemental Figs. S7–S13). In keeping with the modest effects on *K*_d_, the T65A mutant did not alter the maximum fraction bound of any RNA compared to WT ProQ^NTD^. These data suggest that T65 makes only small contributions to RNA binding despite its partial conservation and positioning adjacent to the essential Y70 residue.

Arginine 62 is located one helical turn away from T65 in helix H3 (Fig. 3A). We were intrigued that among the six FinO-domain proteins with solved structures and/or natural RNA ligands identified, *Ec* ProQ is the only one that contains arginine in this position in the concave-face pocket (Supplemental Fig. S1). While arginine is present in homologous positions of ProQs from related enterobacteria (Smirnov et al. 2016; Olejniczak and Storz 2017; Pandey et al. 2020), it is absent from the equivalent position of Lp RocC, which suggested that R62 could be a good candidate for an RNA contact that is different in *Ec* ProQ than *Lp* RocC. Indeed, gel shift data showed that the R62A mutation had a strongly detrimental effect on the binding of all seven RNAs, as the maximum fraction bound was below 40% for most RNAs (Table 1; Fig. 3; Supplemental Figs. S7–S13). The mutant protein’s interaction with *cspE*-3′ was the strongest of all the RNAs with a *K*_d_ value only fourfold weaker than WT ProQ^NTD^, although with a fraction bound just above 40% at the highest concentration tested of R62A ProQ^NTD^ (Table 1). The maximum fractions bound observed for other RNAs were lower still—around 25% for *malM*-3′ and SibA, and below 20% for *cspE*81-3′ and *gapA*-3′—while no binding was detected for RybB and SibB. Although there seems to be a range of effects of R62A on different RNAs, these differential effects are impossible to precisely quantify because of the overall strongly weakened binding. Overall, the R62A mutation is strongly detrimental to the binding of all seven RNAs, although not to the same degree as mutations of Y70 or R80.

We next turned our attention to two basic residues that were found to be important for in vivo ProQ–RNA interactions and were hypothesized to interact with the double-helical portion of a terminator hairpin (Pandey et al. 2020). These residues—arginine 58 and lysine 54—are located one or two additional helical turn(s) further along H3 from R62 on the concave face of ProQ (Fig. 3A). We first analyzed the effects of an alanine substitution of the R58 residue, the closer of the two residues to Y70, and found that an R58A mutation was strongly deleterious for binding of all seven RNAs. As with R80A, no binding of any RNA was detected up to concentrations of 1 µM of the R58A mutant (Table 1; Fig. 3; Supplemental Figs. S7–S13), demonstrating an essential role of R58 in RNA binding by the ProQ FinO domain. On the other hand, the effects of a K54A mutation were more moderate: the maximum fraction bound only reached about 60% for *cspE*-3′, 30% for *malM*-3′, and *cspE*81-3′, and <20% for *gapA*-3′ and RybB, while no binding was detected for SibA and SibB (Table 1; Fig. 3; Supplemental Figs. S7–S13). These effects are comparable to those of the R62A mutation described above. Together, these data show that residues K54 and R58 on helix H3 of the FinO domain play important roles in RNA binding.

### RNA ligands differ in their sensitivity to amino acid substitutions on the periphery

In addition to the central part of the concave-face pocket explored above, our previous B3H studies indicated that some residues on the periphery of the concave face, such as lysine 35 and aspartate 41, could also contribute to RNA binding (Pandey et al. 2020). Interestingly, B3H studies showed that the K35A mutation had more detrimental influence on the binding of SibB than *cspE*, which suggests that K35 could contribute differentially to the binding of distinct RNAs (Pandey et al. 2020). When the binding of our panel of seven RNAs was tested in vitro with the K35A ProQ^NTD^ mutant, there were indeed large differences in the maximum fraction bound reached by each RNA. While the maximum fraction of *cspE*-3′ and *cspE*81-3′ bound to the K35A mutant remained similar to that of WT ProQ^NTD^, the K35A mutant only reached ∼70% RNA bound with *malM*-3′, and 35%–50% with *gapA*-3′, SibA and RybB. The effects on SibB were the strongest, with no binding detected up to 1 µM protein. For several RNAs that reached a much lower fraction bound with K35A than for WT ProQ^NTD^, the *K*_d_ values calculated from the fits were still within three- to fourfold of the *K*_d_ for WT ProQ^NTD^. That the differences in binding were more apparent from the maximum fractions bound rather than the *K*_d_ values could suggest that complexes formed by the K35A mutant with some RNAs were less stable during electrophoresis in native gels, leading to the underestimation of calculated *K*_d_ values. Alternatively, such a result could be explained if the tight binding of some RNAs required a conformational change that was only efficiently induced by ProQ^NTD^ in the presence of K35. Importantly, the relative effects of the K35A mutation on the binding of *cspE*-3′ and SibB are in agreement with the results obtained by the B3H assay in vivo, which also showed a stronger negative effect of this mutation on the binding of SibB (Pandey et al. 2020).

**FIGURE 4.**
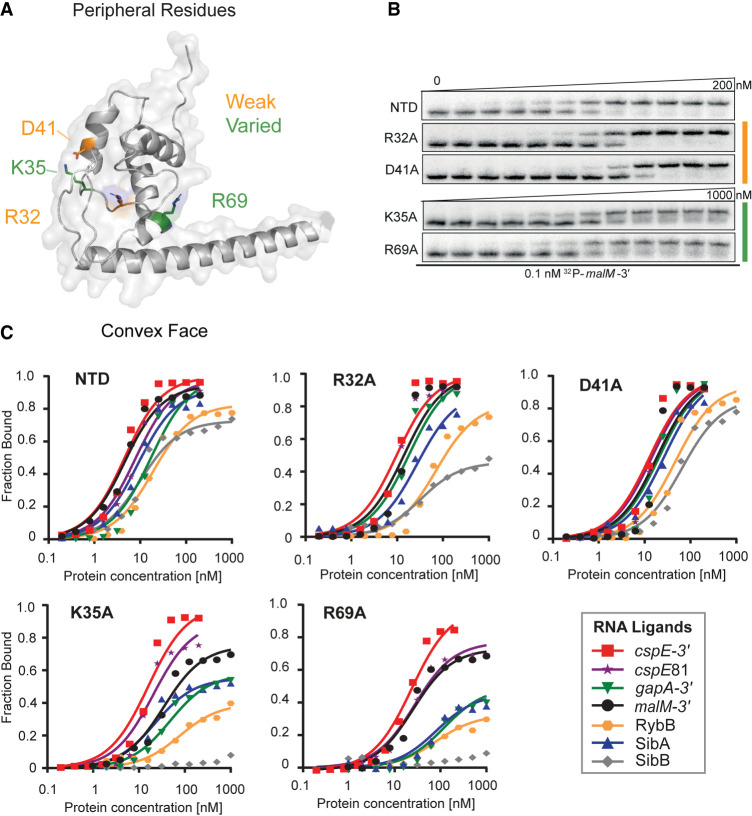
Mutations on the periphery of the central pocket have differential effects on the binding of seven RNAs. (*A*) The location of substitutions on the AlphaFold model of ProQ^NTD^ (Varadi et al. 2022). (*B*) The raw gel data for 1 nM ^32^P-labeled *malM*-3′ binding to the five proteins. (*C*) The plots of fraction bound versus protein concentration for the binding of *cspE*-3′, *cspE*81-3′, *gapA*-3′, *malM*-3′, RybB, SibA, and SibB RNAs to WT ProQ^NTD^, and R32A, D41A, K35A, and R69A mutants measured using the gel shift assay. The data sets in which the maximum fraction bound was above 40% were analyzed by fitting to the quadratic equation. Raw gel data for all RNAs are presented in Supplemental Figures S7–S13. The obtained *K*_d_ values are shown in Table 1.

We next examined the effects of mutating aspartate 41, another residue located at the base of helix H2 (Fig. 4A) that was suggested by previous B3H results to be important for RNA binding (Pandey et al. 2020). In vitro binding data showed that the D41A substitution does not affect the *K*_d_ value of ProQ^NTD^ for *gapA*-3′, but does have a detrimental effect on the binding affinity for other RNAs; the size of its effect ranged from approximately twofold (*cspE*81-3′ and RybB) or threefold (*cspE*-3′ and *malM*-3′) to more than fivefold in the strongest cases (SibA and SibB) (Table 1; Fig. 4; Supplemental Figs. S7–S13). Interestingly, the binding of all seven RNAs to the D41A mutant reached the same maximum fraction bound as with WT ProQ^NTD^ (Table 1), in contrast to the effects of K35A above. In summary, both D41 and K35 contribute modestly to RNA binding by ProQ in vitro, with K35 having more varied effects across RNAs, perhaps mediated through kinetics or facilitating conformational changes in the RNA.

Next, we moved further away from the concave-face pocket of ProQ, and asked whether positively charged amino acids on the convex face of the FinO domain could also affect RNA binding. We first examined the effects of arginine 32, which is conserved either as arginine or lysine in other FinO-domain proteins (Supplemental Fig. S1). In vitro binding data showed that an R32A mutation does not affect the *K*_d_ value of ProQ binding to *gapA*-3′ and *malM*-3′ and has only modest two- to threefold detrimental effects on the binding of *cspE*-3′, *cspE*81-3′, RybB, and SibA (Table 1; Fig. 4; Supplemental Figs. S7–S13). The only RNA that was markedly affected by this mutation was SibB; while its *K*_d_ value was only weakened approximately threefold by the R32A mutation, its maximum fraction bound was reduced to just above 40% (Table 1). The fact that the R32A substitution has only small effects on the binding of most of the RNAs tested suggests that R32 does not make a universal key contact with RNA.

Another basic side chain exposed on the convex face of the FinO domain is arginine 69. Unlike R32, R69 is in an evolutionarily variable position, but its location next to Y70 in the primary amino acid sequence suggested it could affect RNA binding (Supplemental Fig. S1). In vitro binding data showed that an R69A substitution had a three- to fivefold detrimental effect on the binding of *malM*-3′ and *cspE-*3′, and an eight- to 10-fold effect on interactions with SibA and *gapA*-3′ (Table 1; Fig. 4; Supplemental Figs. S7–S13). The R69A mutation had an even stronger detrimental effect on the binding of other RNAs: the maximum fraction bound reached by RybB was below 40%, while the binding of SibB was not detected. The large range of effects of the R69A mutation was reminiscent of the effects of K35A, though perhaps qualitatively slightly stronger for each RNA. It is interesting to note that the rank-order of effects caused by K35A and R69A mutations on the maximum fraction bound reached by each RNA were similar, with *cspE*-3′ being the least affected by either mutation and SibB being the most affected (Table 1).

### The terminator hairpin drives the differential dependence of *cspE*-3′ and SibB on R69

**FIGURE 5.**
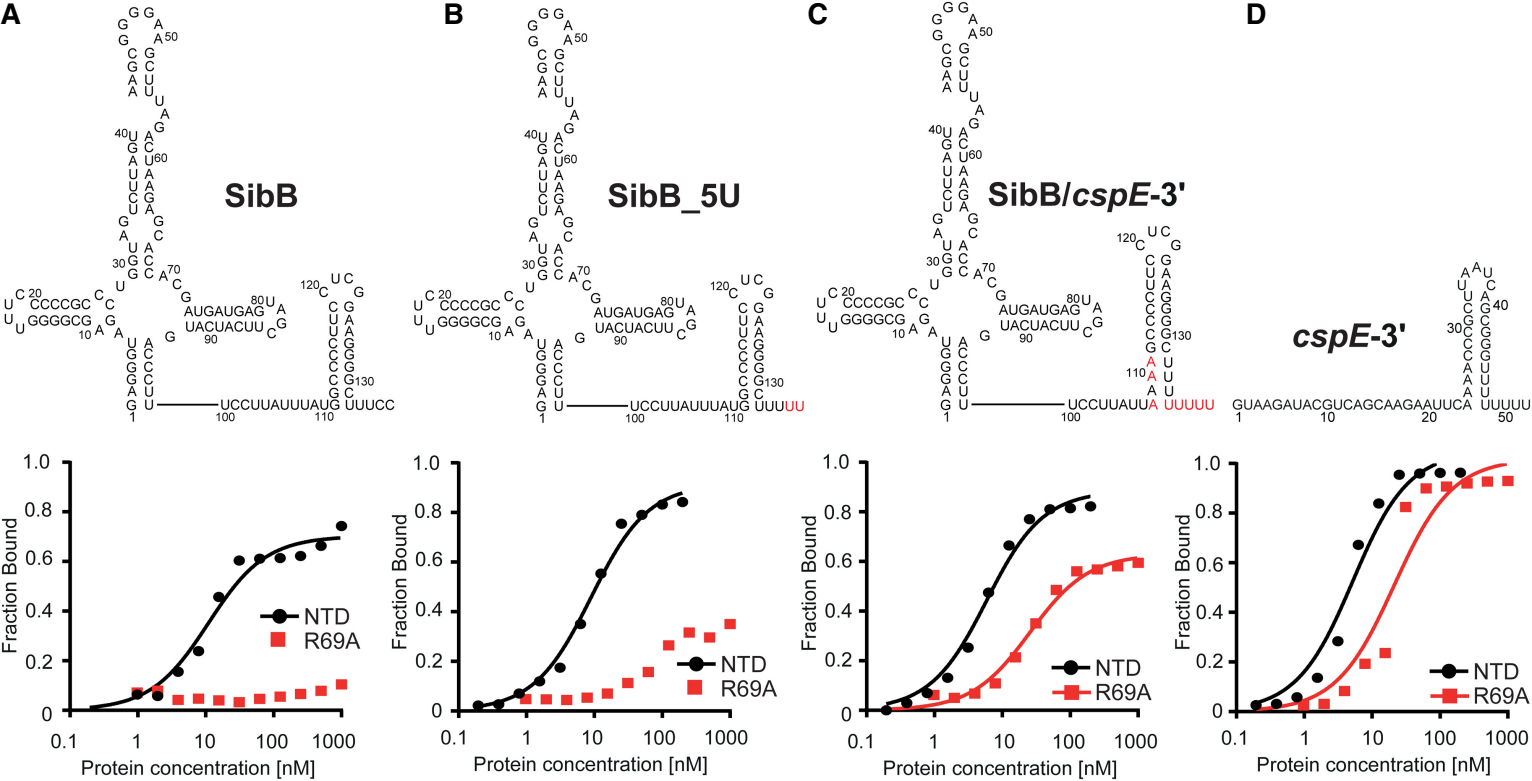
The sequence and structure of the lower part of the terminator hairpins explain differences in the binding of ProQ^NTD^ and its mutants by *cspE*-3′ and SibB RNAs. (*A*–*D*), The secondary structures of RNA molecules are shown *above* the corresponding plots of fraction-bound data for WT ProQ^NTD^ (NTD) or its R69A mutant. The RNA secondary structures were predicted using *RNAStructure* software (Reuter and Mathews 2010). The changes introduced into the sequence of SibB are marked in red font. The data sets in which the maximum fraction bound was above 40% were analyzed by fitting to the quadratic equation. Data for SibB and *cspE*-3′ are the same as in Figure 3. The obtained *K*_d_ values are shown in Table 2, and raw gel data are presented in Supplemental Figure S15.

The results above indicated that several residues, especially K35 and R69, made differential contributions to the binding of the seven RNAs we tested. To explore the basis for these differences, we focused on *cspE*-3′ and SibB RNAs, because the differential effect of a K35A mutation on ProQ binding to these two RNAs was also observed in vivo (Pandey et al. 2020), and because they fell on the opposite ends of the spectrum of susceptibility to ProQ^NTD^ mutants, which increased the likelihood of identifying specific binding determinants. Because the FinO domain of ProQ is known to specifically recognize the intrinsic terminators of its RNA ligands (Holmqvist et al. 2018; Melamed et al. 2020; Stein et al. 2020), we focused on this region of *cspE*-3′ and SibB. The two most striking differences between the terminator regions in these two RNAs are that SibB possesses a shorter oligoU tail that ends with two cytidines and that the base of *cspE*’s terminator hairpin contains several A–U base pairs missing from SibB (Fig. 5). We introduced changes into SibB, the weaker binding RNA, to make its 3′ terminator more similar to *cspE*-3′ and compared the binding of these mutant RNAs to WT and R69A ProQ^NTD^. The first SibB mutant (SibB-5U) replaced the two terminal cytidines with uridines (Fig. 5A,B), resulting in an RNA with a total of five U’s in the tail, four of which are predicted to be single-stranded. While the SibB-5U mutation had no effect on the binding of WT ProQ^NTD^, it improved binding to the R69A mutant, with the mutant protein reaching a fraction bound of >30% when binding was undetectable for unmutated SibB RNA (Table 2; Fig. 5B; Supplemental Fig. S15). We next introduced additional mutations to make the double-stranded base of the SibB terminator hairpin more like that of *cspE*-3′. This SibB–*cspE* chimera now had four A–U base pairs at the base of the terminator and an extended 8-uridine 3′ tail to match that of *cspE*-3′ (Fig. 5C,D). While the mutations in the SibB–*cspE* chimera had only slight effects on the binding of WT ProQ^NTD^, they were sufficient to substantially restore RNA binding to the R69A mutant, which reached a fraction bound of 60% with a *K*_d_ value of 31 nM (Table 2; Fig. 5C; Supplemental Fig. S15). These are considerable improvements from the original SibB RNA, which showed no detectable binding to the R69A mutant, though they do not represent as strong of a binding as seen with *cspE*-3′ RNA, which reached a maximum fraction bound of 88% to the R69A mutant with a *K*_d_ of 20 nM.

**TABLE 2.**
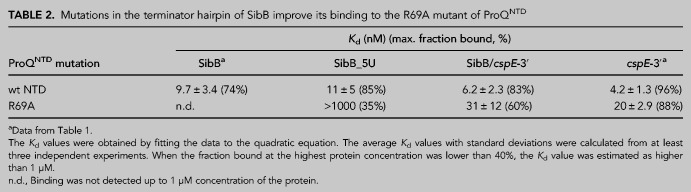
Mutations in the terminator hairpin of SibB improve its binding to the R69A mutant of ProQ^NTD^

### K35 and R69 indirectly contribute to RNA binding at the central RNA-binding site

**FIGURE 6.**
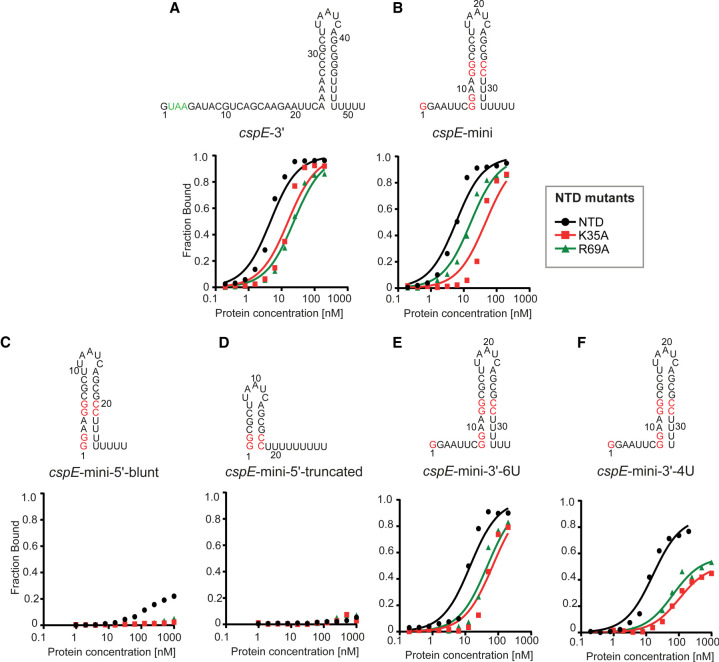
The substitutions of peripheral residues K35 and R69 have indirect effects on the RNA binding in the central pocket of the ProQ^NTD^. The RNA secondary structures were predicted using *RNAstructure* software (Reuter and Mathews 2010). The data sets in which the maximum fraction bound was above 40% were analyzed by fitting to the quadratic equation. Data for *cspE*-3′ are the same as in Figures 3 and 4. The average equilibrium dissociation constant (*K*_d_) values are shown in Table 3, and raw gel data are presented in Supplemental Figure S16.

We hypothesized that the differential effects of R69A and K35A on the binding of ProQ^NTD^ to distinct RNAs could result either from disruptions of direct contacts formed by K35 and R69 with specific RNAs or from indirect effects on alterations of contacts with other amino acids. Because R69 and K35 are located outside of the central concave-face pocket that has been proposed to interact with dsRNA (Pandey et al. 2020; Kim et al. 2022), we wondered whether R69 and K35 could form contacts with single-stranded regions on the 5′ and 3′ side of the terminator hairpin, respectively. We reasoned that if K35 or R69 directly contacted a single-stranded region outside of the hairpin, a shortened RNA would show reduced affinity with WT ProQ, and the mutant protein (K35A or R69A) would show reduced affinity with full-length RNA because it would be sufficient to remove only one of the elements taking part in the interaction to disrupt the contact that they formed. However, no additional loss of affinity would be observed when the mutant protein bound the shortened RNA because this contact would have already been disrupted when either element was removed. Alternatively, if the interaction between the mutant protein and shortened RNA resulted in additional loss of binding energy, the additive effects of these mutations would suggest that they arose from distinct contacts. To conduct these double-mutant experiments, we designed model RNA constructs derived from *cspE*-3′ and compared the effects of their 5′ and 3′ truncations on binding to WT ProQ^NTD^ and its R69A and K35A mutants (Table 3; Fig. 6; Supplemental Fig. S16). First, we constructed a model RNA (*cspE*-mini) by removing the minimally structured 17-nt sequence from its 5′ end and introducing guanosines in three locations within this model construct to enable efficient in vitro transcription of further 5′ truncated constructs—at the 5′ end, the base of the terminator hairpin, and above the A–U base pairs at the base of the hairpin (Fig. 6A,B). The *cspE*-mini construct was found to bind WT ProQ^NTD^ with the same affinity as unmodified *cspE*-3′ (Table 3; Fig. 6B), suggesting that the 17-nt stretch of nucleotides on the 5′ end of *cspE*-3′ does not markedly contribute to RNA binding by WT ProQ^NTD^. Similarly, the loss of these 5′-terminal nt did not decrease the affinity of the R69A variant. Interestingly, however, the *cspE*-mini RNA bound to the K35A mutant with an approximately twofold weakened binding affinity (Table 3; Fig. 6B; Supplemental Fig. S16), suggesting that loss of a longer 5′ single-stranded tail makes RNA binding more dependent on the K35 side chain. While there were only modest effects of removing the first 17 5′ nt, removal of the remaining single-stranded sequence on the 5′ side (Fig. 6C) had a dramatic effect on binding across the board: the resulting *cspE*-mini-5′-blunt construct only achieved about 20% fraction bound with WT ProQ^NTD^ and resulted in no detectable binding to either R69A or K35A mutants (Table 3; Fig. 6C; Supplemental Fig. S16). To explore if releasing the 3′-oligoU tail of *cspE-*3′ would compensate for the contacts to ProQ^NTD^ lost on the 5′ of the hairpin, we further removed four adenosines opposite the 3′-oligoU tail (*cspE*-mini-5′-truncated) (Fig. 6D; Table 3; Supplemental Fig. S16). Despite its longer single-stranded oligoU tail, no binding of this construct was detected even for WT ProQ^NTD^. Together, these data show that single-stranded nucleotides 5′-adjacent to the terminator hairpin are essential for the RNA binding by ProQ^NTD^, while nucleotides further upstream of the terminator hairpin do not markedly affect RNA binding to WT ProQ but may make additional contacts to the protein that help compensate for the loss of K35. Importantly, these data are not consistent with either R69 or K35 directly contacting the 5′ single-stranded RNA, since substitution of either residue led to additional loss of interaction of 5′-truncated RNA.

**TABLE 3.**
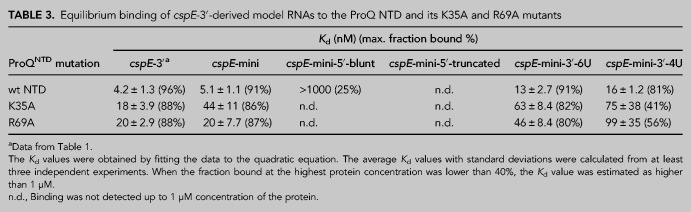
Equilibrium binding of *cspE*-3′-derived model RNAs to the ProQ NTD and its K35A and R69A mutants

To explore whether R69 or K35 could affect RNA binding via contacts with the 3′ oligoU tail, we designed another construct (*cspE*-mini-3′-6U), the 3′ tail of which was shortened from eight to six Us, only two of which are predicted to be single-stranded (Fig. 6E). This 3′ truncation weakened the binding of WT ProQ^NTD^ an additional 2.5-fold beyond *cspE*-mini (Table 3), confirming previous observations that the length of the 3′ U-tail is important for the RNA binding to the FinO domain (Stein et al. 2020). The 3′ truncation also further reduced the binding affinity of both R69A and K35A mutants (by ∼2.4- and ∼1.4-fold, respectively), suggesting that neither of these residues primarily functions by contacting the terminal portion of the 3′ U-tail.

We next examined the effects of a more extensive 3′ truncation, removing an additional two terminal uridines to create *cspE*-mini-3′-4U (Fig. 6F). Binding of all three ProQ^NTD^ variants (WT, R69A, and K35A) was further weakened to this RNA construct, with strong effects seen on the maximum fraction bound for R69A and K35A proteins (Table 3). The fact that substitution of either R69 or K35 had additional detrimental effects on the binding of *cspE*-mini-3′-4U beyond those of *cspE*-mini-3′-6U suggests that these two nucleotides do not directly contact K35 or R69. We note that the effect of the 4-uridine truncation in *cspE*-mini-3′-4U is smaller than previously observed for a different *cspE*-3′ construct with the same truncation (Stein et al. 2020). While the affinities of these two constructs indeed differ in side-by-side binding assays (Supplemental Fig. S17), the result that a 4-uridine truncation is detrimental for RNA binding is consistent across both constructs.

In summary, these mutational studies suggest that R69 and K35 both likely contribute to contacts to the core terminator hairpin of the RNA rather than the adjacent 5′ or 3′ sequences. This is consistent with the above observation that the heightened susceptibility of SibB to R69A and K35A mutations is explained by the properties of its terminator hairpin (Table 2; Fig. 5). In addition, K35 may contribute in part to contacts that become especially important when RNAs possess a shorter extension on the 5′ side of their terminator hairpin.

## DISCUSSION

In this study, we set out to refine our model of molecular recognition of RNA ligands by the FinO domain of *E. coli* ProQ. Data collected from both biochemical and genetic experiments support a model in which all residues of the FinO domain of ProQ involved in RNA binding are located on the concave face of this domain. This includes basic and aromatic side chains poised in the concave-face pocket to interact with intrinsic terminators at the 3′ end of many of ProQ's RNA ligands. Our data suggest that additional residues on the periphery of the concave face—and even pointing toward the opposing convex face of the FinO domain—make smaller contributions to RNA binding that are more varied across RNA ligands. Together, these findings strengthen and extend our prior results, showing that nearly all the ProQ residues implicated previously in RNA binding through in vivo methods indeed impact RNA binding directly in vitro, and allowing for nuanced comparisons to be drawn across a variety of RNA ligands.

### Revisiting the structure of *E. coli* ProQ's FinO domain

Comparison of available structural models of FinO-domain proteins showed that the sidechain of a universally conserved arginine (R80 in *Ec* ProQ) that is critical for RNA binding (Pandey et al. 2020; El Mouali et al. 2021; Rizvanovic et al. 2021) was exposed on the convex face in the *Ec* ProQ NMR structure (Gonzalez et al. 2017), while it pointed toward the concave surface in other proteins (Ghetu et al. 2000; Chaulk et al. 2010; Immer et al. 2020; Kim et al. 2022). Because a convex-face position of this arginine in *Ec* ProQ would be difficult to reconcile with the proposed role of the concave face as the RNA-binding site, we elected to take an unbiased approach to elucidate the in vivo position of R80 in *Ec* ProQ. A compensatory mutagenesis screen identified several amino acid substitutions that partially rescue the detrimental effects of an R80K substitution. The positions of these compensatory substitutions are most consistent with a structural model in which the more conserved face of the FinO domain of *Ec* ProQ possesses a concave pocket containing both R80 and Y70, analogous to the concave-face pocket seen in other structural homologs (Supplemental Fig. S2) as well as in the AlphaFold model for *Ec* ProQ. For this reason, we used the structure of *E. coli* ProQ predicted by AlphaFold for downstream structural modeling in this work (Jumper et al. 2021; Varadi et al. 2022). With respect to why the position of R80 in *E. coli* ProQ could be different inside the cell and in the AlphaFold model than it was in the initially reported NMR structure (Gonzalez et al. 2017), it is possible that the region of the protein containing R80 could be conformationally dynamic in the absence of RNA and that the conformation with a concave-facing R80 residue is locked in place once the protein binds to RNA.

The AlphaFold model provides an explanation for why the seemingly conservative R80K substitution is so strongly deleterious to RNA binding in vivo and in vitro (Figs. 1B,C, 3). While both lysine and arginine are positively charged at physiological pH, the aliphatic portion of lysine's side chain is shorter than that of arginine. If the side chain of R80 points through to the concave face from the β-hairpin containing the residue (Fig. 2E; Supplemental Fig. S5), substitution with lysine could impact RNA binding either directly by moving the residue's charged terminus away from the RNA, or indirectly by destabilizing the hydrophobic core of the protein. Several of the validated compensatory substitutions could subtly alter the hydrophobic environment surrounding R80 to create space for, or help to reposition, lysine's primary amine. Importantly, even the strongest of the identified compensatory substitutions (L91V and V74K) lead to only modest recovery of RNA binding by the R80K ProQ mutant. This underscores the key role that R80 plays in FinO-domain structures, consistent with its universal conservation.

### Structural model of *E. coli* ProQ–RNA interactions

**FIGURE 7.**
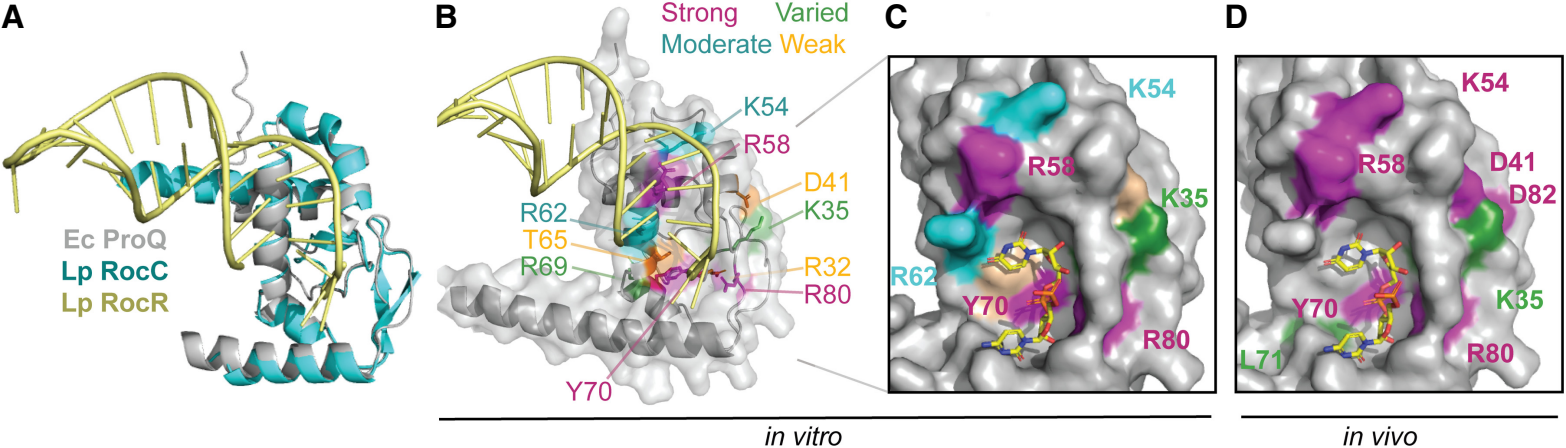
Model for ProQ–RNA interaction. (*A*) Alignment of AlphaFold model of FinO domain of ProQ (Jumper et al. 2021; Varadi et al. 2022) and *Lp* RocC/RocR cocrystal structure (PDB ID: 7RGU; Kim et al. 2022). (*B*) Summary of in vitro biochemical results. Residues which were modified for gel shift experiments are colored based on the size of the detrimental effects on RNA binding when modified: strong (R80, Y70, R58; magenta), moderate (K35, K54, R62; cyan), effects that varied across RNA ligands (K35, R69; green), and those with only weak effects (D41, T65; orange). (*C*,*D*) Surface representation of the concave-face binding pocket of ProQ, with residues colored as in (*B*). The terminal two pyrimidine nucleotides from RocR are shown in stick representation and are colored by atomic identity. The two panels compare (*C*) ProQ residues identified as important for RNA interactions in vitro in the current work and (*D*) residues found in previous B3H experiments to contribute to *cspE*-3′ and SibB binding in vivo (Pandey et al. 2020).

As noted above, the AlphaFold structural model for *E. coli* ProQ shares many structural features with the other four FinO-domain homologs with solved structures (Supplemental Fig. S2; Ghetu et al. 2000; Chaulk et al. 2010; Immer et al. 2020; Kim et al. 2022). Only one structure solved to date has captured high-resolution details of the FinO domain in complex with an RNA ligand: the recent crystal structure of *L. pneumophila* RocC bound to the terminator hairpin of the RocR RNA (Kim et al. 2022). This structure, which shows RocC residues making multiple contacts with the 3′ side of a dsRNA duplex and a 3′-terminal single-stranded tail, offers several important insights for the interpretation of the genetic and biochemical data presented here. The AlphaFold model of *Ec* ProQ^NTD^ aligns very well with the structure of RocC (RMSD = 1.09Å; Fig. 7A), providing a possible structural model for *Ec* ProQ interacting with an RNA terminator hairpin (Fig. 7B). This alignment-based model is consistent with the residues we have identified as most important for RNA binding: K54, R58, R62, Y70, and R80 are all found in the immediate vicinity of the terminator hairpin (Fig. 7B); indeed, each of these residues is conserved in RocC apart from R62 (Supplemental Fig. 1). The fact that the shape of the concave pocket is almost identical between *Lp* RocC and *Ec* ProQ suggests that *Ec* ProQ may also bind the 3′ terminal two nucleotides in this pocket in the vicinity of Y70, R80, and G37 (Fig. 7C,D; Supplemental Fig. S18A). Interestingly, a G37A substitution was previously identified as deleterious to *Ec* ProQ binding in an in vivo forward genetic screen (Pandey et al. 2020). In addition, RNA binding by the F′ FinO protein has been shown to be negatively affected by phosphorylation or oxidation of the 3′-terminal hydroxyl group (Arthur et al. 2011), consistent with an important role of the 3′ terminus in RNA recognition by FinO-domain proteins.

Figures 7C and 7D summarize the effects of substitutions on the concave face of *Ec* ProQ^NTD^, comparing results from in vitro and in vivo binding assays (Figs. 3, 4; Pandey et al. 2020). Overall, there is good alignment between in vivo and in vitro results, suggesting that most residues identified through forward and reverse genetics exert their effects on RNA binding directly, rather than through downstream effects like recruitment of another protein or complex.

### Residues with uniform contributions to interaction with the examined RNAs

While several residues on the concave face of *Ec* ProQ were previously found to be important for the binding of two RNAs (the 3′-UTR of *cspE* and the sRNA SibB; Pandey et al. 2020), the data presented here furthers these observations by examining the effects of substitutions in a purified in vitro system and against a larger set of seven RNAs (Table 1; Figs. 3, 4; Supplemental Fig. S6). The fact that similar conclusions were reached for this larger set of RNAs suggests that many of the RNA ligands of *Ec* ProQ share a similar mode of binding as was seen in the prior genetic study (Pandey et al. 2020) and a RIP-seq analysis of RNAs bound to ProQ R80A mutants (El Mouali et al. 2021). Indeed, most residues examined in this work had similar effects on all seven RNAs tested—whether strong (R58, Y70, and R80), moderate (K54, R62) or weak (R32, D41, and T65) (Table 1; Figs. 3, 4), suggesting that these residues likely recognize core RNA features that all seven RNA ligands have in common.

The tyrosine in position 70 of *E. coli* ProQ is one of the most highly conserved residues in FinO-domain structures and has been previously implicated as critical for RNA binding (Attaiech et al. 2016; Immer et al. 2020; Pandey et al. 2020; El Mouali et al. 2021). Even a conservative substitution from tyrosine to phenylalanine eliminates RNA binding in vitro (Table 1; Fig. 3) and in vivo (Pandey et al. 2020), underscoring the essential role of this hydroxyl group on the concave face. Finally, both neighboring amino acids—R69 and L71—have been found to contribute to RNA binding in ProQ or its homologs (R69: Table 1, Fig. 4) (L71: Attaiech et al. 2016; Pandey et al. 2020; El Mouali et al. 2021; Rizvanovic et al. 2021). The proximity in the primary sequence suggests that these residues could influence RNA binding through effects on the position of Y70.

The other universally conserved amino acid residue that binds the 3′ terminus of the RNA in the RocC/R cocrystal structure is *E. coli* R80. Our work shows that the substitution of this arginine with either lysine or alanine severely impairs RNA binding in vitro (Table 1; Fig. 3) and in vivo (Fig. 1; Pandey et al. 2020). The homologous arginine residue in *Lp* Lpp1663 underwent chemical shift changes in NMR experiments in the presence of RaiZ or oligoU RNA (Immer et al. 2020; Kim et al. 2022). There is also considerable genetic evidence for the importance of this residue: multiple substitutions at this position (serine, histidine, and cysteine) were identified in *Ec* ProQ in an unbiased B3H genetic screen (Pandey et al. 2020) and, indeed, no other residue was found to restore any detectable interaction at this position (Supplemental Table S1). In addition, two independent genetic screens looking for mutations abrogating the role of ProQ in gene expression regulation in *S. enterica* isolated substitutions of R80 to histidine, serine or glycine (El Mouali et al. 2021) or to histidine (Rizvanovic et al. 2021). Importantly, a RIP-seq analysis showed that R80A mutation was detrimental for the binding of numerous RNAs in vivo (El Mouali et al. 2021), which is consistent with its uniformly important role in RNA binding by ProQ observed for seven different RNAs here. Additionally, recent in vivo studies found that RNA-binding deficient mutants such as R80A were subject to degradation by Lon protease, which showed that RNA binding is important for ProQ stability (El Mouali et al. 2021; Rizvanovic et al. 2021).

The double-stranded stem of the RNA terminator hairpin is contacted in the *Lp* RocC cocrystal structure by residues within the H3 helix of the FinO domain (Kim et al. 2022). Alignment of the *Ec* ProQ AlphaFold model (Fig. 7A) places the terminator hairpin's major groove and phosphate backbone in the vicinity of *Ec* H3 residues K54, R58, and R62 (Supplemental Fig. S18B), each of which makes moderate or strong contributions to RNA binding in vitro (Figs. 3, 7B,C). Of these H3 residues, we found substitution of R58 to be especially damaging to RNA interactions (Table 1; Fig. 3), as it was in the B3H assay (Pandey et al. 2020). Additionally, the equivalent residue in *Lp* RocC was found to interact with a phosphate group in the double-stranded helix of RocR (Kim et al. 2022), while the corresponding position of the FinO protein crosslinked with FinP RNA (Ghetu et al. 2000), suggesting that this arginine residue is universally important for RNA binding. The other H3 residues examined here (K54 and R62) are less conserved (Supplemental Fig. S1; Smirnov et al. 2016; Olejniczak and Storz 2017; Pandey et al. 2020) and make smaller contributions to RNA binding in vitro (Table 1; Fig. 3). The importance of K54 is supported by the fact that an alanine substitution of K54 in *Ec* ProQ was found to be detrimental to RNA binding in the B3H assay (Pandey et al. 2020). Additionally, a lysine in the position in *Lp* RocC corresponding to *Ec* K54 is part of the proposed N-cap motif that recognizes the double-stranded portion of the RNA hairpin (Kim et al. 2022). On the other hand, the potential role of R62 in RNA binding has been previously indicated by experiments that showed that a cysteine introduced to the position corresponding to R62 in the F′ FinO protein crosslinked to RNA (Ghetu et al. 2002). Although this residue is not conserved in *Lp* RocC, its position in the alignment-based model suggests it may be able to interact with the double-stranded stem of the terminator hairpin (Fig. 7B; Supplemental Fig. S18B).

### Residues with variable RNA-binding contributions across examined RNAs

In contrast to most residues examined, substitutions of K35 and R69 stand out as having especially varied effects on RNA binding (Table 1; Figs. 3, 4; Supplemental Figs. S7–S13). K35 and R69 are positioned on the concave face and the edge of the convex face, respectively, and occupy evolutionarily variable positions within stretches of highly conserved sequence (Supplemental Figs. S1, S2). The differential contribution of *Ec* K35 to interaction with *cspE*-3′ and SibB has been previously observed in the B3H assay (Pandey et al. 2020), but contributions of *Ec* K35 and R69 have yet to be examined in other homologs. Given that the side chain of R69 points toward the convex face of *E. coli* ProQ, it is quite interesting that its substitution so strongly affects the binding of some of the RNAs in our panel. Given the proximity of R69 to Y70, one possibility is that R69 exerts some of its differential effects on RNA binding through fine-tuning of the environment of Y70. Similarly, the substitution of K35, which is close in sequence to G37, could impact RNA binding indirectly through other residues within the RNA-binding pocket.

Across all ProQ^NTD^ mutants examined, *cspE*-3′ was consistently one of the strongest-binding RNAs and SibB one of the weakest (Table 1; Figs. 3, 4). This in vitro difference in binding strength is consistent with the previous in vivo observation that a K35A mutation in *Ec* ProQ^ΔCTD^ had a strong effect on the binding of SibB in the B3H assay, but only weakly affected the binding of the *cspE* 3′-UTR (Pandey et al. 2020). Tighter interaction of *cspE*-3′ RNA than SibB is also consistent with transcriptome-wide data sets probing in vivo interactions with WT ProQ^NTD^. For instance, *cspE*-3′ was consistently among the top 15 RNAs with 3′ terminators identified as ProQ ligands in *E. coli* using either RIL-seq or CLIP-seq and in *S. enterica* using CLIP-seq (Holmqvist et al. 2018; Melamed et al. 2020; Stein et al. 2020). On the other hand, SibB fell outside of the top 30 RNAs identified by CLIP-seq in *E. coli* and was not present among the top 50 RNAs in data sets from *E. coli* RIL-seq or *S. enterica* CLIP-seq experiments (Holmqvist et al. 2018; Melamed et al. 2020; Stein et al. 2020).

Given the differences in binding to K35A and R69A ProQ^NTD^ mutants observed across the seven RNAs, it is interesting to consider what features of the RNA ligands may drive these differences (Supplemental Fig. S6). A previous study showed that the optimal binding of *malM*-3′ and *cspE*-3′ requires a terminal hairpin stem with a single-stranded tail of at least four uridines (Stein et al. 2020). The data presented here suggest that the structure of the terminator hairpin and the sequences surrounding it modulate the sensitivity of RNA binding to mutants with K35 and R69 substitutions in the FinO domain (Table 2; Fig. 5; Supplemental Fig. S15). For instance, replacing the two terminal cytidines of SibB with uridines markedly improved the binding of the resulting RNA molecule (Table 2; Fig. 5), while shortening the region on either the 5′ or 3′ side of the terminator within a minimal *cspE*-3′ RNA weakened its interaction with ProQ (Table 3; Fig. 6; Supplemental Fig. S16). However, the analysis of either mutant protein binding to minimal *cspE*-3′ RNAs was not consistent with K35 or R69 directly contacting RNA on either side of the terminator hairpin, since substitutions at these residues led to additional binding defects beyond those caused by RNA truncations in these regions (Table 3; Fig. 6). Hence, it is more likely that these residues exert their effects on RNA interaction by modulating the way the amino acids of the primary RNA-binding site contact the base of the terminator stem and the 3′ U-tail.

In summary, the work presented here significantly extends our understanding of how the concave face of the *E. coli* ProQ's FinO domain recognizes RNA ligands. Our data suggest that residues within the concave pocket and along H3 play critical roles in binding for all RNA ligands investigated, and that an RNA's terminator hairpin structure and the nucleotides surrounding it dictate the strength of that RNA's interaction with ProQ^NTD^ and susceptibility to mutations.

## MATERIALS AND METHODS

### Bacterial three-hybrid (B3H) assay

*Escherichia coli* strains, plasmids and oligonucleotides used in the B3H assay are listed in Supplemental Tables S2–S4. NEB5α, purchased from New England Biolabs, was the recipient strain for all cloned B3H plasmids. KB473 served as the reporter strain for all β-gal assays. Each strain and plasmid has specific antibiotic resistance gene(s), listed with the following abbreviations: AmpR (ampicillin and carbenicillin), CmR (chloramphenicol), KanR (kanamycin), StrR (streptomycin), and TetR (tetracycline). All strains were stored as glycerol stocks at −80°C.

In this B3H system, plasmids express three-hybrid components: (i) a DNA–RNA adapter protein, CI-MS2^CP^, tethers (ii) a Bait RNA construct upstream of a test promoter such that it is available for interaction with (iii) an RNAP-tethered prey protein (Fig. 1A). Reporter cells encode a *lacZ* reporter gene downstream from a test promoter on a single-copy F′ episome. Transformation of reporter cells with all three plasmids (pPrey, pAdapter, and pBait) leads to a boost in β-gal levels relative to basal levels indicated by three negative controls in which half of each hybrid component is left out (Fig. 1B); the strength of an RNA–protein interaction correlates to the fold-stimulation in β-gal activity over basal levels when all components are present (Fig. 1C; Wang et al. 2021; Stockert et al. 2022).

### β-galactosidase assays

Liquid assays were performed with the use of the B3H system. Reporter cells (KB473) were cotransformed with pAC-, pBR-, and pCDF-derived plasmids. pAC constructs express the CI-MS2^CP^ fusion protein, while pCDF-pBAD constructs express the MS2^hp^ fusion RNA and pBR-α expresses the α-ProQ fusion protein. For each transformation, there were three negative controls, one where each of these core plasmids was replaced with an “empty” construct. Single colonies from each transformation were inoculated into 1 mL of LB broth supplemented with 0.2% arabinose and antibiotics: carbenicillin (100 µg/mL), chloramphenicol (25 µg/mL), kanamycin (50 µg/mL), and spectinomycin (100 µg/mL) in a 2 mL 96 well deep-well block (VWR) sealed with breathable film (VWR) and shaken at 900 rpm and 37°C overnight. Overnight cultures were back-diluted (1:40) into 200 µL LB supplemented with the same antibiotics and arabinose as outlined above, as well as 0 µM, 5 µM, or 50 µM IPTG (isopropyl-β-d-thiogalactoside; see figure legends) into optically clear 200 µL flat-bottom 96-well plates covered with plastic lids (Olympus). Mid-log cells (OD_600_ 0.3–0.6) were transferred into a new 96-well plate with rLysozyme and PopCulture reagent (EMD Millipore) and allowed to lyse for 0.5–4 h. Lysate was transferred into a fresh optically clear 96 well plate (Olympus) with Z-buffer, ONPG (O-nitrophenyl-β-d-galactopyranoside), and β-mercaptoethanol. β-gal activity was measured by taking OD_420_ values every minute at 28°C for 1 h using a microplate spectrophotometer (Molecular Devices SpectraMax). OD_420_ readings were normalized using the OD_600_ values from directly before lysis to give β-gal activity in Miller units (Thibodeau et al. 2004; Stockert et al. 2022). β-gal activity was averaged over three replicates for each experimental condition and then divided by the highest relevant negative control to give the fold-stimulation. Error for fold was propagated from the standard deviations of experimental and negative-control averages. Assays were conducted in biological triplicate on at least three separate days.

For qualitative plate-based assays, 3.2 µL of mid-log cells from above were pipetted from the 96-well plate onto a large LB agar plate supplemented with inducers (0.2% arabinose and 1.5 µM IPTG), antibiotics (carbenicillin [100 µg/mL], chloramphenicol [25 µg/mL], kanamycin [50 µg/mL], and spectinomycin [100 µg/mL]) and indicators (X-gal [40 µg/mL] and TPEG [200 µM]). Plates were incubated at 37°C overnight, moved to 4°C for at least 1 d, and then photographed with oblique lighting on a black velvet background (Stockert et al. 2022). Images were split into red (R), green (G), and blue (B) channels in ImageJ (Schneider et al. 2012), and 96-well grids were generated with MicroArray Profiler 2012 (Dougherty and Rasband 2012). The intensity of each bacterial patch was measured in each of the three channels (R,B,G), as previously implemented in quantifying β-gal activity using X-gal cytochemical staining (Shlush et al. 2011; Lozano-Gerona and Garcia-Otin 2018). Pure white contains equal intensities in each channel, whereas the blue appearance of X-gal patches correlates with increased intensities in the blue and green channels relative to the red. The “color intensity” of each patch, plotted in Supplemental Figure S4, was calculated by dividing the intensity in the blue channel by the intensity in the red channel (Blue/Red), representing the skew toward blue relative to pure white. Two-tailed, unpaired Student’s *t*-tests were conducted to assess whether values for each mutant were significantly different from those of R80K.

### Western blots

Cell lysates from β-gal assays were normalized based on OD600 with LB plus PopCulture Reagent. Lysates were mixed with 6× Laemmli loading dye, boiled for 10 min at 95°C and electrophoresed on 10%–20% Tris–glycine gels (Thermo Fisher) in 1× NuPAGE MES Running Buffer (Thermo Fisher). Proteins were transferred to PVDF membranes (Bio-Rad) using a semidry transfer system (Bio-Rad Trans-blot Semidry and Turbo Transfer System) according to the manufacturer’s instructions. Membranes were probed with 1:10,000 primary antibody anti-ProQ overnight at 4°C and then a horseradish peroxidase (HRP)-conjugated secondary antibody (anti-rabbit IgG; 1:10,000). Chemiluminescent signal was detected using ECL Plus Western blot detection reagents (Bio-Rad) and a c600 imaging system (Azure) according to the manufacturer’s instructions.

### Library construction for saturation mutagenesis

Single-site mutagenesis was performed using Q5 site-directed mutagenesis. Mutagenic forward and reverse primers were designed using NEBaseChanger. For the Y70X and R80X library plasmids, a region of the primer representing the single codon of interest was replaced by a mixture of 25% of each nucleobase in each of the three sites, written as “NNN” in the primer sequence (Supplemental Table S4). Otherwise, the primers were complementary to the backbone sequence. For multisite saturation mutagenesis of the R80K vector, multiple forward primers were designed to mutagenize one codon position at a time with a degenerate NNN codon (Supplemental Fig. S4A). For each of the three libraries targeting a different 6–7aa region of ProQ, 6–7 forward primers were used in a pooled polymerase chain reaction (PCR) with a single reverse primer. Primers were used in a PCR with 2× Phusion Master Mix (New England Biolabs) and the appropriate parent plasmid: pKB955 (pBrα-ProQ^ΔCTD^) for Y70X and R80X libraries and pSP144 (pBrα-ProQ^ΔCTD^-R80K) for the R80K multisite compensatory libraries. For Y70X and R80X libraries, PCR products were Kinase–Ligase–DpnI (KLD) treated in 10 µL reactions containing 1 µL PCR Product, 1 µL T4 DNA Ligase (New England Biolabs), 1 µL T4 DNA Ligase buffer (New England Biolabs), 1 µL T4 polynucleotide kinase (PNK; New England Biolabs), 1 µL DpnI (New England Biolabs), and 5 µL MilliQ water. For the multisite-ProQ-R80K libraries, a commercial KLD mixture was used according to the manufacturer’s instructions (New England Biolabs). KLD products for each library were transformed into NEB5α cells. Cells were serially diluted and spread on LB-carbenicillin and incubated at 37°C overnight to provide a near-lawn for library preparation and to allow for estimates of colony numbers (Supplemental Table S5). Plasmid for each library was directly miniprepped from a cell slurry harvested from the overnight plates and stored at −20°C for further use.

### B3H screening

Screens of these library plasmids were performed with the use of the B3H assay. The plasmid libraries were transformed into KB473 reporter cells pretransformed pKB1210 (pBait-*malM*-3′UTR) and pCW17 (pAdapter). Cells were heat-shocked and plated on LB agar supplemented with inducers (0.2% arabinose and 1.5 µM IPTG), antibiotics (carbenicillin [100 µg/mL], chloramphenicol [25 µg/mL], kanamycin [50 µg/mL], and spectinomycin [100 µg/mL]) and indicators (X-gal [40 µg/mL] and TPEG [200 µM]). Plates were incubated at 37°C overnight. Each time that a transformation was performed, positive and negative controls were transformed alongside the experimental conditions to allow for comparison of blue/white levels (positive: wild-type ProQ; negative: alpha empty, pBrα or pSP144, ProQ-R80K). The plates were transferred from the 37°C incubator to 4°C once colonies had grown to sufficient size, ∼18 h. Plates were stored at 4°C for a minimum of ∼5 h and a maximum of ∼3 d before being examined for the presence of blue colonies. Colonies that showed any blue color above that of pPrey-empty negative-control colonies were restruck on indicator plates to ensure single blue colonies. Plasmid from these colonies were miniprepped and sent for sequencing. Sequences were aligned to the sequence of wild-type *E. coli* ProQ to identify mutations at the codon of interest.

### Preparation of overexpression constructs and protein purification

The sequences of *E. coli* FinO amino-terminal domain (NTD; residues 1–130) of ProQ were cloned into pET15b vector (Novagen), as described (Stein et al. 2020). In the construct, the coding sequence of the protein was preceded by His_6_-tag and TEV protease recognition sequence (ENLYFQ↓S), so an additional serine residue remains after the cleavage of the amino-terminal His_6_-tag. The mutated variants of NTD of ProQ were obtained by site-directed mutagenesis, where the substitutions were introduced by specifically designed primers (Supplemental Tables S6, S7) to change a single amino acid in the protein sequence. All constructs were overexpressed in BL21 Δ*hfq* strain (a kind gift of Prof. Agnieszka Szalewska-Pałasz, University of Gdańsk), and purified as described (Stein et al. 2020). The samples were stored in buffer (50 mM Tris, pH 7.5, 300 mM NaCl, 10% glycerol, and 1 mM EDTA) at −80°C as 10 and 20 µL aliquots and used without refreezing. The concentration of proteins was determined by measuring the absorption at 280 nm using an extinction coefficient of 9650 M^−1^ cm^−1^.

### RNA preparation

The DNA templates used for in vitro transcription were obtained by Taq polymerase extension of chemically synthesized overlapping oligodeoxyribonucleotides (Sigma-Aldrich, Metabion, Supplemental Table S8). RNA molecules were transcribed with T7 RNA polymerase and purified using denaturing gel electrophoresis, as described (Milligan et al. 1987; Olejniczak 2011). In the next step, RNAs were 5′-^32^P labeled using T4 polynucleotide kinase (Thermo Scientific), followed by phenol–chloroform extraction, purification on denaturing gel and precipitation with ethanol.

### In vitro RNA-binding (gel shift) assay

Prior to use, RNA molecules were denatured for 2 min at 90°C followed by 5 min refolding on ice. The concentration series of all proteins was prepared by twofold dilutions from the highest concentration (given above the gel image). In each binding reaction, 1 nM ^32^P-labeled RNA was mixed with a protein sample diluted in binding buffer (25 mM Tris, pH 7.5, 150 mM NaCl, 5% glycerol, 1 mM MgCl_2_), and incubated for 30 min at RT in low-protein binding microplates pretreated with a solution containing 0.0025% bovine serum albumin (BSA). After this time, reactions were loaded onto a 6% polyacrylamide gel (19:1) running in 0.5× TBE buffer at 4°C. After the electrophoresis, gels were vacuum-dried and exposed to phosphor screens overnight. The signal was quantified using a phosphorimager and MultiGauge software (Fuji FLA-5000), data were fitted to a quadratic equation using GraphPad Prism software, and the equilibrium dissociation constant (*K*_d_) values were calculated as described (Stein et al. 2020). The average *K*_d_ values given in the tables were calculated based on at least three independent experiments.

## SUPPLEMENTAL MATERIAL

Supplemental material is available for this article.

## Figures and Tables

**Ewa Stein:**
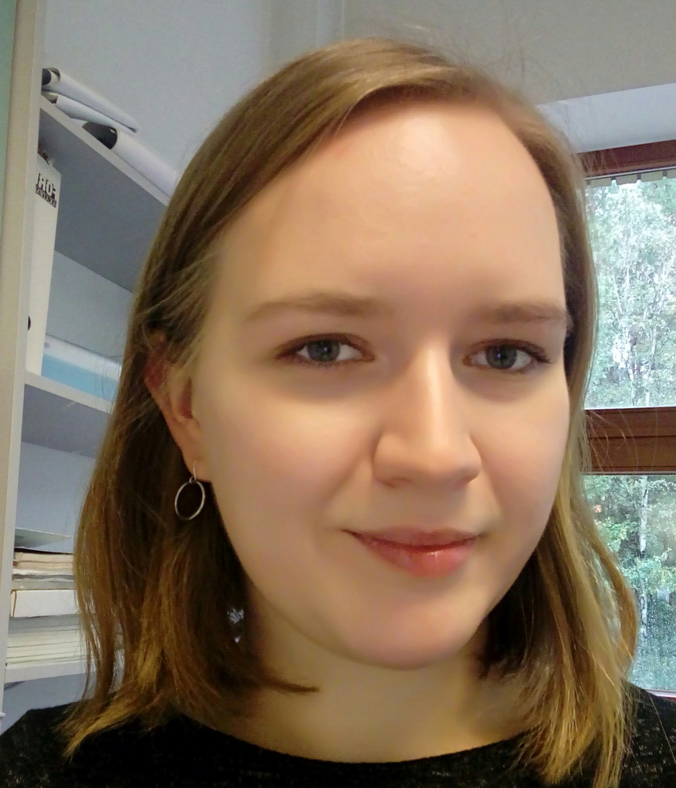


## MEET THE FIRST AUTHOR

***Meet the First Author(s)* is an editorial feature within *RNA*, in which the first author(s) of research-based papers in each issue have the opportunity to introduce themselves and their work to readers of *RNA* and the RNA research community. Ewa Stein is the first author of this paper, “Biochemical and genetic dissection of the RNA-binding surface of the FinO domain of *Escherichia coli* ProQ.” She carried out this work as a graduate student in Mikołaj Olejniczak's laboratory in the Institute of Molecular Biology and Biotechnology at Adam Mickiewicz University in Poznan. Her research was focused on studying the interactions between bacterial protein ProQ and its RNA partners. Recently, she joined the group of Kinga Kamieniarz-Gdula in the Institute of Molecular Biology and Biotechnology at Adam Mickiewicz University as a research assistant in a project concerning alternative polyadenylation in mammalian cells.**

**What are the major results described in your paper and how do they impact this branch of the field?**

Using a set of *E. coli* ProQ protein mutants, we found that substitutions of K35 and R69 residues, evolutionarily variable but adjacent to conserved ones, showed varied effects on the binding of different RNA ligands. We confirmed in vitro the relevance of RNA-binding residues initially identified in vivo, such as R58, Y70, and R80. Our data suggest that those three residues form the main RNA-binding site of ProQ protein, while additional contacts make smaller contributions to RNA binding and are more varied across RNAs. Combining in vitro and in vivo approaches together with our collaborator Professor Katherine Berry from Mount Holyoke College, we propose a model in which all residues within the FinO domain of ProQ protein involved in RNA binding are located on the concave face of this domain.

**What led you to study RNA or this aspect of RNA science?**

I joined the laboratory primarily interested in the interactions between short RNAs and RNA-binding proteins in bacteria, which I found incredibly interesting and nuanced. Such interactions affect many aspects of bacterial metabolism, and understanding the underlying mechanisms may be crucial in developing novel antibiotics.

**What are some of the landmark moments that provoked your interest in science or your development as a scientist?**

I have been interested in biology in general since high school, and particularly in molecular biology since one lesson at biology class when I found out about molecular tools used in criminology. Later during my studies, I attended a lecture given by Tom Cech during his visit to Poznań. That meeting was very inspiring, and it was then that I began to seriously consider a career in science.

**If you were able to give one piece of advice to your younger self, what would that be?**

Learn to rest and work on maintaining a balance between work and life. The best solutions to problems come while running.
